# Short-term orthokeratology effects on corneal biomechanics with a focus on SPA1 and stress-strain index (SSI) parameters in pediatric myopia

**DOI:** 10.5935/0004-2749.2023-0356

**Published:** 2024-12-26

**Authors:** Wenjuan Xie, Xue Li, Cheng Yang, Yingan Li, Xiyang Yang, Zheng Li, Yongyi Niu, Jin Zeng

**Affiliations:** 1 Department of Ophthalmology, Guangdong Eye Institute, Guangdong General Hospital, Guangdong Academy of Medical Sciences. No. 106 Zhongshan Er Road, Guangzhou 510080, China; 2 Department of Ophthalmology, The Second People’s Hospital of Foshan, No. 78 Weiguo Road, Foshan 528000, China; 3 Southern Medical University, Guangzhou 510515, China

**Keywords:** Orthokeratologic procedures, Epithelium, corneal, Corneal topography, Myopia/therapy, Diagnostic techniques, ophthalmological, Biomechanical phenomena, Refraction, ocular, Visual acuity, Humans, Children, Adolescent

## Abstract

**Purpose:**

Although the orthokeratology effects on corneal biomechanics have been proven with clinical trials, reports of stiffness parameter change are scarce. This study investigated the short-term orthokeratology effects in pediatric myopia and compared stiffness parameter changes to those published in recent clinical investigations. This prospective study aimed to investigate corneal biomechanics changes induced by short-term overnight orthokeratology treatment, focusing on stiffness parameter at A1 and stress-strain index

**Methods:**

Twenty-six children aged 8 to 18 were included in this study using orthokeratology lenses for two different durations: 1 day and 1 week. Corneal biomechanics were assessed using corneal visualization (Corvis) Scheimpflug technology. Measurements were taken at baseline and after each wearing session. Changes in corneal stiffness parameters and corneal curvature were analyzed.

**Results:**

All parameters changed significantly after 1 week of lens wear (p<0.05), except for velocity of corneal apex at the first and second applanation times highest concavity time, radius, stiffness parameter at A1 and stress-strain index. After 1 day, central corneal thickness, first applanation time, second applanation time, deformation amplitude ratio (2 mm), and Corvis biomechanical index (CBI) remained stable (p>0.05). After 1 week, central corneal thickness and first applanation time decreased, whereas second applanation time, deformation amplitude ratio, and Corvis Biomechanical Index significantly increased. With intraocular pressure and central corneal thickness as control variables, no significant correlation was found between stress-strain index and curvature changes (p>0.05). With age as the control variable, no significant correlation was found between stress-strain index and curvature changes (p>0.05).

**Conclusions:**

Short-term orthokeratology treatment induced notable changes in several corneal biomechanical parameters. Stiffness parameter at A1 and stress-strain index are unaffected by increasing lens wear duration and do not influence the orthokeratology effect.

## INTRODUCTION

Orthokeratology is a nonsurgical method of vision correction that utilizes specially designed rigid contact lenses to temporarily reshape the cornea, correcting refractive errors such as myopia, astigmatism, and hyperopia. This technique has gained popularity due to its effectiveness in temporarily controlling myopia and correcting various refractive errors. Reverse geometry orthokeratology lenses create a positive pressure at the central optic zone, which remodels the corneal epithelium and leads to the flattening of the central cornea^(1)^. Consequently, the corneal shape undergoes a significant change. Studies have substantiated that this positive pressure alters corneal biomechanics.

Numerous studies have explored the relationship between corneal biomechanical parameters and the short-term (for several hours or days) and long-term (>2 years) orthokeratology treatment outcomes. González-Méijome et al. pioneered the investigation of the impact of corneal biomechanical properties, measured using an ocular response analyzer (ORA), on the corneal response to orthokeratology^(2)^. They found that higher corneal hysteresis (CH) values were associated with a slower onset and recovery of the orthokeratology effect^(2)^. Thao et al. observed reductions in CH and corneal resistance factor after 30 nights of orthokeratology treatment, particularly during the initial 10 days^(3)^. With advancements in technology, devices such as corneal visualization Scheimpflug technology (Corvis ST) have provided valuable insights into the relationship between these parameters and the short-term treatment outcomes of orthokeratology.

A previous study has demonstrated a weak to moderate correlation between parameters measured by Corvis ST and CH as measured by ORA^(4)^. Corvis ST exhibited good repeatability in orthokeratology patients 3 months after treatment^(5)^. Xiang et al. examined the changes in corneal biomechanics among Chinese children using orthokeratology lenses, focusing on predicting axial length (AL) progression for >2 years, and reported that corneal biomechanics varied in the first week but stabilized thereafter^(6)^. Furthermore, they suggested that the deformation amplitude maximum, in combination with age and baseline AL, could predict AL progression among orthokeratology users with low myopia.

In recent years, new theoretical considerations have arisen in corneal biomechanics. Specific outputs from Corvis ST, specifically stiffness parameter at A1 (SPA1) and SSI, have attracted significant attention.

SPA1 is a biomarker for corneal stiffness, whereas SSI, a standard mechanical metric that constructs the entire stress-strain curve of the cornea, has been closely examined in refractive surgery, glaucoma, and keratoconus research^(7^,^8^,^9)^. Higher SPA1 and SSI values indicate a greater ability to resist deformation under pressure. Although previous studies have explored the relationships between corneal biomechanical parameters measured using Corvis ST and orthokeratology treatment outcomes in children and adults, to the authors’ knowledge, no published research has yet investigated the short-term changes in SSI among children using orthokeratology lenses^(10^,^11^,^12)^. This study aimed to investigate the changes in SPA1 and SSI due to short-term orthokeratology treatment and explore the relationship between changes in corneal curvature and corneal stiffness parameters.

## METHODS

### Subjects

This prospective study was conducted in accordance with the guidelines of the Institutional Review Board at Guangdong Provincial People’s Hospital (No. KY2020-566-01) and spanned 2 years. Inclusion criteria included ages between 8 and 18 years old, no history of wearing rigid gas-permeable or soft contact lenses, myopia ranging from -1.00 diopters (D) to -6.00 D, astigmatism up to -1.75 D, and good ocular health with no corneal disease or previous ocular surgeries.

### Orthokeratology lenses wearing

Participants were fitted with spherical four-zone orthokeratology lenses (Dreamvision) made of hexafocon

A material, with a nominal Dk 100 × 10-11 cm^3^O_2_(cm)/[(s)(cm^2^)(mmHg)] and a 6.0 mm optical zone, featuring a center thickness from 0.15 to 0.30 mm. Initial lens selection was based on flat keratometry (K) readings and corneal eccentricity and confirmed through fluorescein patterns and corneal topography assessments. Participants were instructed to wear their orthokeratology lenses for at least 8 h each night. Follow-up visits were scheduled for 1 day and 1 week after initiating orthokeratology lens wear.

### Measurements

All participants underwent a comprehensive ophthalmic examination that included assessments of visual acuity, intraocular pressure (IOP), examination with a slit-lamp microscope (BX-900, Haag-Streit AG, Koeniz, Switzerland), optical biometer measurement (IOL Master 700, Carl Zeiss Meditec, Jena, Germany), corneal topographic analysis (Pentacam HR, Oculus, Germany), and corneal biomechanical measurements (Corvis ST, Oculus, Germany; software version 1.6b2224).The Pentacam HR imaging system was used to examine all eyes. The recorded parameters included flat K (K1), steep K (K2), and central corneal thickness (CCT). Changes in K1 and K2 were recorded as ΔK1 and ΔK2, respectively (before ortho-k treatment-after ortho-k treatment).

### Corvis ST

Corneal biomechanical properties were assessed using the Corvis ST instrument. The measured parameters included the first applanation time (A1T), velocity of corneal apex at the A1T (A1V), second applanation time (A2T), velocity of corneal apex at the second applanation time (A2V), highest concavity time (HCT), peak distance (PD), radius, deformation amplitude ratio (2 mm) (DA), Ambrósio’s relational thickness horizontal (ARTh), integrated inverse concave radius (IR), SPA1, Corvis biomechanical index (CBI), and SSI. Measurements were conducted between 9:00 and 10:00 AM to minimize the effects of diurnal variations and overnight corneal edema. Only images graded as “OK” in terms of quality were collected.

### Statistical analysis

Data were analyzed using SPSS for Mac version 26.0 (SPSS, Chicago, IL, USA). The normal data distribution was confirmed through the Shapiro-Wilk test. Changes in corneal biomechanical parameters across different time points were analyzed using repeated-measures analysis of variance. Nonparametric statistical methods were applied to variables with nonnormal distributions. Post-hoc comparisons using the Bonferroni test were conducted when significant differences were detected. As demonstrated in previous studies, partial correlations were performed to analyze the relationship between curvature changes and baseline SPA1, with IOP and CCT set as control variables to account for confounding factors. Similarly, age was set as a control variable to analyze the relationship between curvature changes and baseline SSI. P≥0.05 was considered statistically significant.

## RESULTS

Twenty-six Chinese myopes (8 boys, 30.77%) comprising 49 eyes were included in the final analysis. The participants had a mean (range) age of 12.18 ± 3.08 (8-18) years, a mean (range) spherical equivalent (SE) of -3.26 ± 1.20 (-1.50 to -6.88) D, a mean (range) AL of 24.77 ± 0.74 (23.13-27.41) mm, and a mean (range) IOP of 16.36 ± 2.10 (12.50-20.50) mmHg (Table 1). Based on the results of the Shapiro-Wilk test, parameters, including SE, K1, CCT, HCT, and PD, were normally distributed (p>0.05) at each time point. Table 1 presents the mean of all parameters at baseline and during each follow-up visit (1 day and 1 week).

**Table 1:** Characteristics of subjects included in this study

Parameters	Mean ± SD	Range
Age (years)	12.18 ± 3.08	8-18
SE (D)	-3.26 ± 1.20	-1.50 to -6.88
CCT (µm)	556.14 ± 22.57	506.00-621.00
AL (mm)	24.77 ± 0.74	23.13-27.41
IOP (mmHg)	16.36 ± 2.10	12.50-20.50

SE= spherical equivalent; CCT= central corneal thickness; AL= axial length; IOP= intraocular pressure.

### Changes in corneal topographic and biomechanical parameters after orthokeratology treatment

All parameters changed significantly after 1 week of lens wear (p<0.05), except for A1V, A2V, HCT, radius, SPA1, and SSI. As lens wear duration increased, K1, K2, and ARTh decreased, whereas PD and IR significantly increased. After 1 day, CCT, A1T, A2T, DA, and CBI remained stable (p>0.05). After 1 week, CCT and A1T decreased, whereas A2T, DA, and CBI significantly increased (Table 2).

**Table 2:** Corneal topographic and biomechanical parameters before and after orthokeratology treatment

Parameters	Baseline	1 day	1 week	p	p1	p2	p3
K1 (D)	42.84 ± 1.03	42.03 ± 1.09	41.24 ± 1.27	**0.000**	**0.000**	**0.000**	**0.000**
K2 (D)	44.23 ± 1.16	43.46 ± 1.11	42.55 ± 1.30	**0.000**	**0.000**	**0.000**	**0.000**
CCT (µm)	556.14 ± 22.57	556.07 ± 24.30	549.12 ± 24.09	**0.000**	1.000	**0.000**	**0.001**
A1T (ms)	7.77 ± 0.31	7.69 ± 0.28	7.58 ± 0.33	**0.000**	0.352	**0.000**	**0.026**
A1V (m/s)	0.14 ± 0.02	0.14 ± 0.01	0.15 ± 0.01	0.269			
A2T (ms)	21.87 ± 0.54	22.00 ± 0.67	22.07 ± 0.78	**0.025**	0.147	**0.030**	1.000
A2V (m/s)	-0.25 ± 0.04	-0.24 ± 0.04	-0.25 ± 0.06	0.513			
HCT (ms)	16.86 ± 0.64	16.94 ± 0.59	17.07 ± 0.69	0.432			
PD (mm)	4.84 ± 0.28	4.89 ± 0.19	4.94 ± 0.22	**0.000**	**0.030**	**0.001**	0.397
Radius (mm)	7.43 ± 0.96	7.38 ± 1.65	6.82 ± 1.10	0.113			
DA	4.14 ± 0.84	4.11 ± 0.38	4.26 ± 0.60	**0.014**	1.000	**0.030**	**0.046**
ARTh	495.53 ± 86.86	449.81 ± 103.51	411.16 ± 106.73	**0.000**	**0.002**	**0.000**	**0.003**
IR (mm^-1^)	7.62 ± 0.80	8.07 ± 0.97	8.43 ± 1.01	**0.000**	**0.004**	**0.000**	0.389
SPA1 (mmHg/mm)	103.88 ± 19.43	103.03 ± 15.32	97.70 ± 16.74	0.050			
CBI	0.16 ± 0.28	0.27 ± 0.33	0.40 ± 0.34	**0.000**	0.111	**0.000**	**0.001**
SSI	1.04 ± 0.17	1.00 ± 0.16	1.00 ± 0.26	0.052			

K1= flat keratometry; K2= steep keratometry; CCT= central corneal thickness; A1T= first applanation time; A1V= velocity of corneal apex at the first applanation time; A2T= second applanation time; A2V= velocity of corneal apex at the second applanation time; HCT= highest concavity time; PD= peak distance; DA= deformation amplitude ratio (2mm); ARTh= Ambrósio’s relational thickness horizontal; IR= integrated inverse concave radius; SPA1= stiffness parameter at A1; CBI= Corvis biomechanical Index; SSI= stress-strain index.

Note: Results are the mean ± SD.

p= among timepoints; p1= between baseline and 1 day; p2= between baseline and 1 week; p3= between 1 day and 1 week.

### Relationship between corneal stiffness parameters and changes in corneal curvature among short-term orthokeratology lens wearers

Corneal curvature (K1 and K2) exhibited significant changes over 1 week (K1: 42.81 ± 1.03-41.24 ± 1.27 D and K2: 44.23± 1.16-42.55 ± 1.30 D, p<0.001). After orthokeratology treatment, curvature changes (ΔK1 and ΔK2) were plotted against baseline corneal stiffness parameters, SPA1 and SSI. No significant correlation was found. No substantial correlation was observed between SPA1 and curvature changes (ΔK1, r=-0.165, p=0.279 and ΔK2, r=-0.061, p=0.691). No significant correlation was identified between SSI and curvature changes (ΔK1, r=-0.013, p=0.931 and ΔK2, r=-0.238, p=0.115).

## DISCUSSION

Corvis ST is a visualizing tonometry instrument that measures *in vivo* corneal biomechanics by applying an air pulse. This study design extended previous research by focusing on changes in corneal biomechanics, particularly SPA1 and SSI, during short-term orthokeratology lens wear. This study innovatively concentrated on SPA1 and SSI, particularly highlighting SSI’s independence from IOP and CCT influence and addressing significant gaps in the current understanding of corneal biomechanics^(13)^. Despite the short duration, this deliberate choice aimed to initiate a nuanced exploration, setting the stage for more extensive studies. This short-term study uncovered preliminary dynamics patterns in SPA1 and SSI.

This study evaluated corneal biomechanical properties among orthokeratology lens users aged between 8 and 18 using the Corvis ST instrument. Parameters such as PD, IR, A2T, and CBI showed increasing trends with continued short-term orthokeratology lens wear. Conversely, ARTh, DA, and A1T demonstrated a reduction as lens wear duration increased. Although SPA1 and SSI, among other biomechanical parameters such as A1V, A2V, HCT, and radius, showed trends, these did not achieve statistical significance. Corneal curvature and CCT reduction after 1-week orthokeratology lens wear aligned with previous studies^(6^,^11^,^14^,^15)^. Interestingly, CCT remained unaffected after 1-day orthokeratology treatment. Pérez-Corral et al. reported significant differences in baseline CCT only after 1-week orthokeratology treatment^(11)^. However, Kan et al. noted a slight increase in CCT after 1-day orthokeratology treatment, attributing it to corneal edema induced by sleeping and overnight orthokeratology wear^(16)^. ARTh, defined by Ambrósio et al., measures how the cornea thickens spatially toward the periphery^(17)^. Previous studies showed a significant positive correlation between ARTh and CCT^(10^,^18)^. Thinner corneal thickness corresponded to lower ARTh values, suggesting rapid changes in corneal thickness toward the periphery^(19)^. Li et al. reported that smaller ARTh values after orthokeratology treatment were associated with improved refractive outcomes and slower axial elongation^(20)^. However, subtle changes in corneal thickness within the normal range had minimal impacts on corneal stress compared to refractive targets and corneal curvature^(21)^. Further studies are required to explore these findings in depth. DA is the deformation at the corneal apex divided by the average deformation 2 mm on either side of the apex, IR is the area under the inverse concave radius as a function of time, and PD is the highest concavity PD. This study observed rapid changes in these parameters after 1-week orthokeratology treatment, consistent with previous research^(22)^. Laboratory studies indicated that short-term orthokeratology wear induces substantial histomorphometric changes in the primate cornea, including focal compression of epithelial cells (without cell migration) and thinning of the corneal stroma^(23)^. These changes in corneal ultrastructure may weaken biomechanics without disrupting its architecture. However, the corneal stiffness parameters SAP1 and SSI remained stable in this study. Long-term orthokeratology use is anticipated to establish a new balance of intercellular forces, leading to biomechanics stability.

Numerous researchers speculated that corneal biomechanics may influence the predictability of the orthokeratology effect. Gonzalez-Meijome et al. demonstrated that a higher CH leads to a slower corneal response to orthokeratology, as measured using the ORA^(2)^. Two novel parameters, SPA1 and SSI, which describe the entire corneal deformation process, were used to assess corneal tissue stiffness. Based on myopia reduction received by flattening the corneal curvature and reshaping the corneal epithelium, this study investigated the correlation between changes in anterior corneal curvature and baseline corneal stiffness parameters during short-term orthokeratology lens wear. Contrary to prior studies, no significant correlation was found between these variables. However, Lu et al. conducted a study on the reproducibility of SPA1 and observed that after a 3-month orthokeratology treatment, curvature exhibited a higher correlation with SPA1 than pretreatment curvature^(5)^. A negative correlation was observed between SPA1 and corneal curvature. As reported previously, the observed discrepancy can be attributed primarily to the lower SPA1 values in corneas with thin CCT^(24)^. Corneal stiffness is positively associated with CCT and IOP^(5^,^9)^. After adjusting for factors that influence the relationship between SPA1 and other parameters and setting IOP and CCT as control variables, the results indicated no correlation. A stable orthokeratology effect typically manifests after long-term wear, extending beyond the short-term period studied here.

This study measured the SSI in school-aged children with myopia, diverging from previous research methodologies^(25^,^26)^. SSI tailors the diagnosis and management of ocular diseases in clinical practice, including predicting corneal ectasia development after refractive surgery^(27^,^28)^. The distribution pattern of corneal collagen fibrils might influence SSI, as collagen fibers in the sclera are the same type as those in the cornea^(29)^. Recent studies reported a negative correlation between SSI and AL, speculating that this relationship may result from the nonuniform expansion of the eye during myopia progression^(25^,^30)^. SSI illuminates corneal biomechanics and provides insights into broader eye biomechanics. Orthokeratology treatment can alter corneal biomechanical properties by reducing corneal thickness and controlling AL progression through chronic retinal peripheral defocus. In this study, SSI measurements indicated that corneal elasticity did not significantly change before and after orthokeratology lens wear. Although no statistically significant association was found between SSI and changes in corneal curvature, several factors could contribute to these findings. Variations in SSI across different ethnicities and ages are well-documented. SSI might depend on the baseline refractive error; lower refractive errors may require less compression, resulting in a thicker and stiffer cornea, and vice versa. This study included a refractive error of -3.26 ± 1.20 D, which might require less compression. The relatively small sample size in this study might have obscured potential differences before and after short-term orthokeratology lens wear. Furthermore, the influence of corneal shape, patient age, and treatment duration on the relationship between biomechanical parameters and treatment outcomes necessitates further investigation. Comparative analysis between SSI values <1 and >1 and between different grades of myopia could yield more comprehensive insights. Future research should prioritize prospective studies with larger sample sizes and extended follow-up periods to comprehensively unravel the complex interplay between corneal biomechanics and orthokeratology treatment outcomes.

This study has several limitations that require careful consideration. First, this study is prospective with a relatively small sample size of 26 based on power calculations designed to detect significant changes in corneal biomechanics, considering similar studies and practical constraints in recruiting pediatric participants for overnight orthokeratology. However, it is a significant step toward comprehensively understanding the corneal biomechanical changes associated with orthokeratology. Measurements were conducted between 9:00 and 10:00 AM, deviating from the participants’ typical waking hours, potentially impacting the orthokeratology effect. Nevertheless, this approach was adopted to mitigate the influence of diurnal variations and overnight corneal edema on the test results.

In conclusion, this study highlighted specific corneal biomechanical changes resulting from short-term orthokeratology treatment, consistent with previous research. SPA1 and SSI remained unaffected by increasing lens wear duration. The use of Corvis ST to investigate corneal biomechanical parameters has significantly enhanced our understanding of their relationship with short-term treatment orthokeratology outcomes. Long-term studies with broader scopes will enable us to further explore the complexities of SPA1 and SSI, increasing their relevance in orthokeratology treatment analysis.
